# The accuracy of the FIT in detecting advanced neoplasm is highest in young people aged 40 to 49 years: an analysis based on sex and age

**DOI:** 10.1007/s00384-023-04470-1

**Published:** 2023-06-26

**Authors:** Kailong Zhao, Shuyuan Wang, Zhen Yuan, Wenwen Pang, Suying Yan, Xinyu Liu, Wanting Wang, Ben Yi, Qiurong Han, Yao Yao, Yanfei Liu, Tianhao Chu, Zhiqiang Feng, Qinghuai Zhang, Xipeng Zhang, Chunze Zhang

**Affiliations:** 1 Department of Colorectal Surgery, Tianjin Union Medical Center, Tianjin, China; 2 School of Medicine, Nankai University, Tianjin, China; 3 School of Integrative Medicine, Tianjin University of Traditional Chinese Medicine, Tianjin, China; 4 Tianjin Medical University, Tianjin, China; 5 Department of Clinical Laboratory, Tianjin Union Medical Center, Tianjin, China; 6 The Institute of Translational Medicine, Tianjin Union Medical Center, Tianjin, China; 7 Tianjin Institute of Coloproctology, Tianjin, China

**Keywords:** Accuracy and efficiency of the faecal occult blood test, Screening, Screening strategy, Faecal immunochemical test (FIT), Screening age, Advanced neoplasm

## Abstract

**Background:**

Colorectal cancer (CRC) is one of the most common cancers and is associated with high incidence and mortality rates worldwide. CRC has caused a tremendous loss of human health and wealth. The incidence and mortality of colorectal carcinoma are increasing in young adults. Early cancer detection and prevention are made possible through screening. At present, the faecal immunochemical test (FIT) is a noninvasive method that can be used for the large-scale clinical screening of CRC status. Therefore, this study, based on CRC screening results in Tianjin from 2012 to 2020, was conducted to analyse the major differences in diagnostic performance parameters according to sex and age.

**Methods:**

This study was based on 39,991 colonoscopies performed for individuals in the Tianjin CRC screening program from 2012 to 2020. Of these individuals, they had complete FIT and colonoscopy results. The differences in FIT results were analysed by sex and age.

**Results:**

According to this study, males were generally more likely to develop advanced neoplasms (ANs) than females, and the prevalence increased with age. Males with negative FIT results were more likely to have advanced neoplasms than females with positive results. The accuracy of the FIT in detecting ANs in each age group was 54.9%, 45.5%, 48.6% and 49.5% in the 40–49, 50–59, 60–69, and ≥ 70 age groups, respectively.

**Conclusions:**

The FIT detected ANs with highest accuracy in the 40–49 age group. Our research can provide guidance to formulate CRC screening strategies.

## Introduction

As the third most common cancer and the second leading cause of cancer-related deaths worldwide [1], colorectal cancer (CRC) caused an estimated 2 million cases of morbidity and 1 million deaths worldwide in 2020 alone [2]. CRC imposes a serious medical and health burden worldwide and in China [3]. According to the latest data, China has a high incidence of CRC [4], and the prevalence among young people (younger than 50 years) is gradually increasing [5, 6]. It is important to improve the implementation of CRC management and screening plans, which can be used to reduce the incidence and mortality associated with CRC. This is particularly true for young people [7]. The guaiac fecal occult blood test (gFOBT) is a common method used to detect colon carcinoma. However, the faecal immunochemical test (FIT) is frequently used as a screening method for CRC [8]. Research results have shown that the FIT is substantially better than the gFOBT [9, 10]. Some trials have shown that the FIT has superior detection rates and compliance for cancer and advanced adenomas compared to the gFOBT [11]. In addition, the quantitative FIT is more flexible than the gFOBT because numerical results are reported, allowing positive threshold customization [12]. Therefore, in this analysis, we focused on the difference in the diagnostic accuracy of the FIT in detecting any advanced neoplasm (AN) in different age and sex groups to provide better strategies for CRC screening programs.

## Methods

### Study population

A database of individuals in Tianjin, China, who screened for colonoscopy between 2012 and 2020 at the medical institution designated by the plan was used. Tianjin’s screening program, which began in 2012, mainly targets residents over 40 years of age. Colonoscopy is recommended for people at high risk for CRC. The risk assessment was based on a family history of a first-degree relative with CRC, adenoma or polyps, which was collected in the form of a questionnaire. Additionally, FIT results and intestinal symptoms were used for risk assessment. For individuals with FIT screening results indicating high risk, the FIT was performed using Abiogene Biotechnology Ltd, with a cut-off value of 100 ng/mL. An annual screening scheme was formulated by the Tianjin CRC Screening Office. The first round was conducted in 2012–2014; the second round was conducted in 2015–2017; and the third round was conducted in 2018–2020. The main screening population included 60- to 74-year-old individuals in the first round, 50- to 60-year-old individuals in the second round, and 40- to 50-year-old individuals in the third round. Screened patients identified as being at high risk for CRC were asked to participate in free colonoscopies as soon as possible, within a week to several weeks. This large cross-sectional study included 89,535 individuals who underwent colonoscopies within the CRC screening program. After excluding patients with missing valid information, there were 39,991 patients with valid records, including complete FIT test results, complete questionnaire content, and colonoscopy results. Before registration, all subjects signed informed consent forms, and all research and methods followed the principles of the Helsinki Declaration. The Tianjin Union Medical Center’s Ethics Committee approved this research.

### Measurement and definition

A team of experienced endoscopists who were certified by the medical institution’s Endoscopy Committee performed all colonoscopies. In accordance with the latest clinical guidelines, gastrointestinal pathologists confirmed all abnormal findings. Only patients with high-quality colonoscopies were included, with adequate bowel preparation, photo documentation of caecal landmarks, and a withdrawal time > 6 min. In addition, based on this study, we divided the patients into two groups according to colonoscopy results: patients with any AN (including CRC and advanced adenoma with a diameter of ≥ 10 mm, villous components or highly atypical hyperplasia) and patients with normal colonoscopy. Normal colonoscopy meant that the colonoscopy results showed no abnormalities or some nonadenomatous benign lesions. Patients were categorized into four groups according to age: 40–49, 50–59, 60–69, and ≥ 70 years. The risk factors included in the multifactor regression analysis were derived from the screening population questionnaires, among which chronic diarrhoea referred to diarrhoea lasting for more than 3 months in the past 2 years, with each attack lasting for more than 1 week. Chronic constipation referred to constipation lasting for more than 2 months every year in the past 2 years. Smoking status was divided into never smoking, current smoking and former smoking. Other factors were dichotomous.

### Data analysis

A chi-square test was used to analyse whether sex and age were statistically significant for FIT results by single-factor analysis. Statistical software was used to calculate various evaluation indicators used to analyse the target results, including the specificity (Sp), sensitivity (Se), positive predictive value (PPV), and negative predictive value (NPV). We used colonoscopy as the gold standard, and by comparing statistical FIT results with colonoscopy results, we could obtain the accuracy of the detection of intestinal lesions by the FIT in all age groups. The accuracy was calculated as (A + B/N) %, where A is the number of patients with positive FIT results and resulting lesions detected by colonoscopy, B is the number of patients with negative FIT results and normal colonoscopy and N is the total number of people in each age group. Multivariate analysis, which consisted of logistic regression analysis, was performed to evaluate the relationship between risk factors and the accuracy of FIT detection; odds ratios (ORs) and 95% confidence intervals (CIs) were calculated. Microsoft Excel software (version 16.46) was used to manage the data, and SPSS software (version 26) was used to analyse the data. The *p* values were two-sided, and *p* < 0.05 was considered statistically significant.

## Results

### Characteristics of the study population

A total of 39,991 people were included in the analysis. Table 1 provides the baseline data for analysis classified by sex and each factor outcome. The distribution of the number of males and females was 18,865 vs 21,126. The average age of the population was 63.04 (7.789) years. A smaller number of participants were aged between 40 and 49 years, accounting for 6.0% of the sample. The number of people aged between 60 and 69 years old accounted for the largest part, approximately 48% of the total sample. The proportion of individuals who had never smoked was substantially higher among females than males (93.1% vs. 58.3%), and considerably more males than females were current smokers (33.6% vs. 5.7%).

**Table 1:** Distribution of sex, age and FIT results among participants

	**Total (*N* = 39991)**	**Males (*N* = 18865)**	**Females(*N* = 21126)**	***P***
**Age at colonoscopy, years (%)**				< 0.01
40–49	2405(6.0)	1194(6.3)	1211(5.7)	
50–59	10,014(25.0)	4328(22.9)	5686(26.9)	
60–69	19,211(48.0)	8998(47.7)	10,213(48.3)	
≥ 70	8361(20.9)	4345(23)	4016(19.0)	
**FIT results (%)**				< 0.01
Positive	21,252(53.1)	10,292(54.6)	10,960(51.9)	
Negative	18,739(46.9)	8573(45.4)	10,166(48.1)	
**Smoking status (%)**				< 0.01
Never	30,705(76.8)	11,042(58.3)	19,663(93.1)	
Current	7548(18.9)	6346(33.6)	1202(5.7)	
Former	1738(4.3)	1477(7.8)	261(1.2)	
**Family history of CRC in an FDR (%)**				< 0.01
No	37,103(92.8)	17,604(93.3)	19,499(92.3)	
Yes	2888(7.2)	1261(6.7)	1627(7.7)	
**History of chronic diarrhoea (%)**				< 0.01
No	33,122(82.8)	15,255(80.9)	17,867(84.6)	
Yes	6869(17.1)	3610(19.1)	3259(15.4)	
**History of chronic constipation (%)**				< 0.01
No	32,742(81.9)	16,258(86.2)	16,484(78.1)	
Yes	7249(18.1)	2607(13.8)	4642(21.9)	
**Colonoscopy findings (%)**				< 0.01
Normal colonoscopy	36,652(91.7)	16,811(89.1)	19,841(93.9)	
Any advanced neoplasm	3339(8.3)	2054(10.9)	1285(6.1)	

*FIT* Faecal Immunochemical Test, *CRC* Colorectal Cancer

### Prevalence of ANs

Statistics show that the prevalence of ANs increases with age. The prevalence rate among males in any age group was higher than that among females in the corresponding age group. As shown in Table 2, since ANs is less common in females, prevalence of ANs in both FIT( −) and FIT( +) among females is lower than the prevalence of ANs in both FIT( −) and FIT( +) among males.

**Table 2:** Prevalence of any advanced neoplasm according to age, sex and FIT results

Age	Females					Males				
Any advanced neoplasm	FIT (-)		FIT ( +)		Total	FIT (-)		FIT ( +)		Total
	N	Prevalence %	N	Prevalence %	Prevalence %	N	Prevalence %	N	Prevalence %	Prevalence %
40–49	23	3.3	7	1.3	2.5	40	5.8	12	2.4	4.4
50–59	100	4.0	128	4.1	4.0	178	9.0	178	7.6	8.2
60–69	246	5.0	384	7.3	6.1	394	9.9	677	13.5	11.9
≥ 70	175	8.7	222	11.1	9.9	212	11.0	363	15.0	13.2
Total	544	5.4	741	6.8	6.1	824	9.6	1230	12.0	10.9

*FIT* Faecal Immunochemical Test

### The FIT accurately detects ANs

Table 3 shows the Se, Sp, PPV and NPV of the FIT for detecting ANs in different age groups by sex. We observed that the Se was the highest for males aged 60–69 years (62.7%), and the Sp was the highest for males aged >  = 70 years (43.6%). Among females, the highest Se (63.0%) was in the 40–49 age group, and the Sp was the highest in the >  = 70 age group (50.0%). We found that for both men and women, the PPV of the FIT for detecting ANs was the lowest (34.8% vs 21.5%) and the NPV was the highest (58.5% vs. 76.8%) in the 40–49 age group. By matching the FIT detection results with the enteroscopy results, we calculated the FIT detection accuracy for different age groups. The results are shown in Table 4. Through comparison, the diagnostic accuracy of the FIT for the 40–49 age group was high compared with that for the other age groups, reaching 54.9%; the diagnostic accuracy of the FIT for the 50–59 age group was the lowest among all age groups at 45.4%.

**Table 3:** FIT accuracy for detecting ANs according to sex and age

Age	(%)			
	Se	Sp	PPV	NPV
Males				
40–49	58.9	34.4	34.8	58.5
50–59	61.4	37.1	53.2	45.1
60–69	62.7	41.8	62.8	51.5
≥ 70	61.4	43.6	64.0	40.9
Females				
40–49	63.0	34.7	21.5	76.8
50–59	62.7	37.6	35.0	65.3
60–69	57.9	44.1	45.0	57.0
≥ 70	57.6	50.0	52.4	55.3

*Se* Sensitivity, *SP* specificity, *NPV* Negative Predictive Value, *PPV* Positive Predictive Value

**Table 4:** Accuracy of the diagnosis of ANs by the FIT in different age groups

Age	FIT result	Any advanced neoplasm		Accuracy percentage
		Yes (N)	No (N)	(%)
40–49	Positive	19	1018	54.9
	Negative	63	1305	
50–59	Positive	306	5192	45.4
	Negative	278	4238	
60–69	Positive	1061	8275	48.6
	Negative	640	9235	
≥ 70	Positive	585	3836	49.5
	Negative	387	3553	

*FIT* Faecal Immunochemical Test

### Correlation between the selected risk factors and accuracy of the FIT

Multivariate logistic regression analysis was performed to determine the factors that affect the accuracy of the FIT. The results are shown in Table 5. A history of chronic diarrhoea (OR 1.378, 95% CI 1.319–1.466; *P* < 0.01), a history of chronic constipation (OR 1.326, 95% CI 1.259–1.397; *P* < 0.01) and a history of CRC in first-degree relatives (OR 2.842, 95% CI 2.615–3.015; *P* < 0.01) were associated with the accuracy of diagnosis by the FIT.

**Table 5:** Multivariate logistic regression analysis of diagnostic accuracy

Factor	P	OR	(OR) 95% CI
History of chronic diarrhoea (vs. no)			
Yes	< 0.01	1.378	1.319–1.466
History of chronic constipation (vs. no)			
Yes	< 0.01	1.326	1.259–1.397
Age at colonoscopy (vs. 40–49 years)			
50–59	< 0.01	0.685	0.625–0.751
60–69	< 0.01	0.786	0.720–0.858
≥ 70	< 0.01	0.836	0.762–0.918
History of intestinal cancer in a first-degree relative (vs. no)			
Yes	< 0.01	2.842	2.615–3.089
Smoking status (vs. never smoking)			
Smoking	< 0.01	0.817	0.773–0.864
Former smoking	< 0.01	0.800	0.723–0.885
Sex (vs. males)			
Females	0.236	0.974	0.932–1.018

*OR* Odds Ratio, *CI* Confidence Interval

## Discussion

In this mass screening, 39,991 people were selected and evaluated based on age and sex. They were classified as having AN and normal colonoscopy according to their colonoscopy results. Through statistical analysis, we obtained the accuracy of the FIT the detection of ANs in each age group and found that the accuracy of the FIT was highest in the 40–49 age group. Moreover, we compared the accuracy of the FIT in the detection of nonadvanced adenoma in the 40–49 age group. In our study, a history of chronic diarrhoea or chronic constipation improved the accuracy of the FIT in the detection of ANs, and having a first-degree relative with a history of CRC also increased the accuracy, with a strong correlation.

In the results of this study, we emphasized the higher accuracy (54.9%) of the FIT in AN detection in subjects aged 40–49 years. In previous studies in the USA, CRC was the second leading cancer and the third leading cancer-related cause of death among people less than 50 years of age. In the past several years, the prevalence of CRC among young people has gradually increased [13–15]. Some research has reported that the prevalence of CRC in the 45–50 age group is already similar to that in the 50–59 age group [16]. Other studies have shown that the number of risk factors for ANs in people under 50 years of age has increased compared with those for people older than 50 years [17, 18]. The reason may be related to the environment, diets and living habits of young people. Existing studies have shown a clear relationship between CRC screening and a decrease in the CRC prevalence among people over age 50. Other studies in the USA showed that the decrease in the incidence rate of CRC among individuals over 50 years of age is related to the increase in the use of colonoscopy, indicating that CRC can be prevented to a large extent through colonoscopy screening [13]. As most of these screening programs target people over 50 years of age, their beneficial effects may not be applicable to younger patients [13]. Therefore, in screening programs, efforts to improve the screening rate of young people will help reduce the risk of CRC.

Our research results provide some screening strategies for the early prevention and detection of CRC or ANs in the population. Compared with most of the current clinical guidelines in China, which suggest that the screening age for CRC begin at 50 years of age [19–21], the US guidelines recommend screening all males and females with average risks from the age of 45 years [22–24]. Europe also continues to recommend CRC screening at the age of 50 years [25], as does the UK. Canada proposes screening between the ages of 50 and 75 years [26]. Australia proposes starting FITs at the age of 50 years [27]. In the Asia–Pacific region, Japan provides annual FIT screening at the age of 40 years. There is no upper age limit [28]. South Korea provides a biennial FIT program from the age of 50 years [29]. We found that most CRC screening starts at age 50 worldwide. Among all screening strategies, the FIT and colonoscopy are the main methods of screening [22, 30–33]. Although the sensitivity of colonoscopy is substantially higher than that of the FIT [34], the compliance of population-based CRC colonoscopy screening is low, and the effect is limited [32, 33, 35]. Therefore, based on the conclusion of our study, the accuracy of the FIT in detecting ANs is high in the 40–49 age group. However, the Se was lower than that in previous studies, which is related to the fact that the participants in our study could only be included if they had positive scores on the screening risk questionnaire or positive FIT tests. Therefore, all the included participants were at high risk for CRC [36]. In addition, combined with the rising prevalence of CRC among young people, it is suggested that initial age of cancer screening be reduced to 45 or 40 years to better achieve the desired screening effect, improve the early detection rate of CRC and better decrease the incidence of late CRC [37]. In addition, the FIT is still recommended as the preferred method in screening programs for young people. The conclusion we obtained from the multivariate regression analysis regarding the accuracy of the FIT in detecting ANs is the conclusion: having chronic constipation, haematochezia, a first-degree relative with a history of CRC, and chronic diarrhoea are related to the high accuracy of FIT in detecting ANs [38].

Our study has some advantages and limitations. First, in this clinical screening study, we evaluated the accuracy of the FIT in detecting ANs in an at-risk cohort. Although the accuracy of the FIT in screening for ANs is only approximately 50%, few studies have evaluated the accuracy by performing the FIT and colonoscopy for all screening subjects. Our research can supplement these results. In addition, this was also a population-based screening study, which enabled us to obtain information about the AN or CRC screening program and further optimize the screening strategies, which is the advantage of our research. However, there are also shortcomings. We focused on the accuracy of the FIT in the 40–49 age group, but most screened people were between the ages of 50 and 69 years, and a smaller number of people aged 40–49 years were included. For this reason, these decisions may not be representative. Moreover, the Se and Sp of the FIT in each age and sex group were lower than the previous results [35], which may be because the subjects in our analysis were screened by the FIT or colorectal cancer risk questionnaire. The included population was at high risk of CRC.

In conclusion, the accuracy of the FIT in detecting ANs in the 40–49 age group was high. The factors related to the accuracy of detection were having a history of chronic constipation, a history of chronic diarrhoea, and a first-degree relative with a history of CRC. With the increasing prevalence of CRC among young people, it is recommended that the age for CRC screening programs be reduced to 45 or 40 years. The FIT has a high accuracy in screening for ANs and, as a noninvasive method, can be recommended as the preferred method for CRC screening in young people.

## Data Availability

Due to restrictions on ethical approval involving patient data and anonymity, the datasets analysed during the current study are not publicly available but can be obtained from the appropriate authors upon reasonable request. If you would like to obtain data from the study, please contact corresponding author Professor Chunze Zhang.
